# Molecular cloning and characterization of pirarucu (*Arapaima gigas*) follicle-stimulating hormone and luteinizing hormone β-subunit cDNAs

**DOI:** 10.1371/journal.pone.0183545

**Published:** 2017-08-28

**Authors:** Thais Sevilhano, Roberto Feitosa de Carvalho, Nélio Alessandro de Jesus Oliveira, João Ezequiel Oliveira, Vinicius Gonçalves Maltarollo, Gustavo Trossini, Riviane Garcez, Paolo Bartolini

**Affiliations:** 1 Biotechnology Department, IPEN-CNEN/SP, Cidade Universitária, São Paulo, SP, Brazil; 2 Department of Pharmacy, Faculty of Pharmaceutical Sciences, University of São Paulo, São Paulo, Brazil; 3 Genetic Ichthyology Laboratory, Bioscience Institute, University of São Paulo, São Paulo, SP, Brazil; Universite de Rouen, FRANCE

## Abstract

The common gonadotrophic hormone α-subunit (GTHα) has been previously isolated by our research group from *A*. *gigas* pituitaries; in the present work the cDNA sequences encoding FSHβ and LHβ subunits have also been isolated from the same species of fish. The FSH β-subunit consists of 126 amino acids with a putative 18 amino acid signal peptide and a 108 amino acid mature peptide, while the LH β-subunit consists of 141 amino acids with a putative 24 amino acid amino acid signal peptide and a 117 amino acid mature peptide. The highest identity, based on the amino acid sequences, was found with the order of Anguilliformes (61%) for FSHβ and of Cypriniformes (76%) for LHβ, followed by Siluriformes, 53% for FSHβ and 75% for LHβ. Interestingly, the identity with the corresponding human amino acid sequences was still remarkable: 45.1% for FSHβ and 51.4% for LHβ. Three dimensional models of ag-FSH and ag-LH, generated by using the crystal structures of h-FSH and h-LH as the respective templates and carried out via comparative modeling and molecular dynamics simulations, suggested the presence of the so-called “seat-belt”, favored by a disulfide bond formed between the 3^rd^ and 12^th^ cysteine in both β-subunits. The sequences found will be used for the biotechnological synthesis of *A*. *gigas* gonadotrophic hormones (ag-FSH and ag-LH). In a first approach, to ascertain that the cloned transcripts allow the expression of the heterodimeric hormones, ag-FSH has been synthesized in human embryonic kidney 293 (HEK293) cells, preliminarily purified and characterized.

## Introduction

The two pituitary gonadotrophic hormones, follicle-stimulating hormone (FSH) and luteinizing hormone (LH), are strictly related to gonadal development and differentiation, regulating reproductive processes such as gametogenesis and follicular growth in all vertebrates, fish included [1–4]. These hormones can thus be employed as spawning inductors to improve breeding and set up reproductive centers, which is particularly important for endangered and overexploited species of fish used for human consumption, like *Arapaima gigas* [5–8].

Gonadotrophic hormones (GTHs) are non-covalently bound heterodimeric glycoproteins composed of a common α-subunit, important for signal transduction, protein folding and heterodimer stabilization, and a hormone-specific β-subunit that influences secretion, disulfide bond pairing, metabolic clearance rate and elicits a particular biological response [9–12]. The folding, secretion, circulatory half-life, maintenance of heterodimer stability and biological potency of these glycoprotein hormones are also influenced by their carbohydrate moiety [13]. The N-linked oligosaccharides of most gonadotrophic hormones from a variety of species differ in branching and, especially, in two terminal modifications: sialylation and sulfation. These greatly influence the half-life of glycohormones, since sialylation protects them against clearance, while sulfation leads to a more rapid clearance rate upon binding to a receptor on the surface of liver endothelial cells [14]. The carbohydrate moiety, besides altering the pharmacokinetics of a given hormone, has also been shown to affect its receptor interactions. Human α-subunit Asn^52^ oligosaccharide, close to the putative receptor-binding region, seems in fact to exert a hormone-specific functional role in the hormone-receptor interaction and receptor activation [15].

As mentioned, the specificity of the hormone action is mostly determined by the β-subunit, but it is also known that determinants in the heterodimer account for the synthesis of hormone-specific carbohydrate structures. The diversity of the oligosaccharide structures finally synthesized indicates that the assembly of the α-subunit with the hormone-specific β-subunit, occurring in the endoplasmic reticulum, elicits a hormone-specific processing of N-linked oligosaccharides [14].

Following the pioneering work of Suzuki et al. [16] reporting the successful purification and characterization of two chemically distinct gonadotropins from chum salmon and the consequent proposal that there also existed two gonadotropins in teleosts, a large number of fish species have had their gonadotrophic hormones cloned and characterized. The cloning of the cDNAs of FSH and the LH α- and β-subunits of these species includes the Atlantic halibut [17], goldfish [18, 19], Russian sturgeon [20], rockfish [21], Manchurian trout [22], zebrafish [23], Senegalese sole [24], marbled eel [25], Chinese sturgeon [26], Atlantic cod [27] and sablefish [28]. Among these, Choi et al. [22], Huang et al. [25], Kim et al.[21] and So et al. [23] also presented phylogenetic trees including the α- and β-subunit of both gonadotropins. With regard to fish FSH and/or LH heterologous expression, of particular interest are the syntheses obtained via baculovirus in silkworm larvae for goldfish [29] and those in the yeast *Pichia pastoris* for Japanese eel [30], tilapia [31,32] and zebrafish [33]. Either stable or transient CHO expression of both gonadotropins was also obtained for Manchurian trout [22] and for zebrafish [23]. With the exception of Kazeto’s group, who synthesized and purified Japanese eel [34] and channel catfish [35] FSH and LH obtained in the S2 drosophila cell line, the synthesized recombinant gonadotropins were not extensively purified and characterized in most of these works, being mainly analyzed via SDS-PAGE and Western blotting and tested in different bioassays. None of the above-mentioned species of fish, moreover, are representative of the order of Osteoglossiformes and not even of the superorder of Osteoglossomorpha, to which *A*. *gigas* belongs.

*Arapaima gigas* (pirarucu) is a giant Arapaimidae native to the Amazon River basin that can reach 3 meters in length and weigh up to 250 kg, present in Brazil, Ecuador, Colombia, Peru and Bolivia. This species is in danger of disappearing because of the exploitation by the fishing industry and increasing human presence [5]. It is largely used for food and extractivism purposes and commercial breeding is still incipient. In previous work, our research group isolated and characterized the cDNA of the α-subunit of *A*. *gigas* gonadotropins encoding for a 115 amino acid glycoprotein with a signal peptide of 24 and a mature peptide of 91 amino acids and also carried out a phylogenetic analysis based on its amino acid sequence [36]. In the present work, the cDNAs of ag-FSHβ and of the ag-LHβ have also been isolated from *A*. *gigas* pituitaries and sequenced for future biotechnological synthesis of these gonadotrophic hormones, useful for physiology and fertility studies and related applications. A three dimensional comparative modeling and a phylogenetic analysis, based on the two different β-subunit transcripts, was also carried out. As a first approach, ag-FSH has also been synthesized in HEK293 cells and preliminarily characterized.

## Materials and methods

The project was approved by the Animal Ethic Commission of our Institution. Licenses were obtained from the Brazilian Institute of Environment (IBAMA), Reg. N° 5198393; Regional Council of Veterinary Sciences, Reg. N° 32242-PJ and Ministry of Fishery and Aquiculture, Reg. N° 00755-SP. Pituitaries were processed and stored in the Tissue Bank of the Genetic Ichtiology Laboratory of the University of Sao Paulo.

### Animals

Six pirarucus (*A*. *gigas*), two males and four females, 2–4 years old, with total length between 100 and 140 cm and weighing 15–40 kg, were obtained in a commercial fishing station in the district of São João da Boa Vista, SP, Brazil (21° 58’ 09” latitude South and 46° 47’ 53” longitude West), to which the remains of the fish carcasses were returned. Pirarucus were caught from small lakes by the station personnel, using appropriate fishing nets. After decapitation, carried out by the same personnel under the supervision of our group and, in particular, of one of the authors belonging to the Genetic Ichtiology Laboratory of the Biological Science Institute of the University of São Paulo, pituitary glands were immediately removed following decapitation, stored in “RNA later solution” (Life Technologies), and transported to São Paulo for the experiments. Pituitaries were processed and stored in the Tissue Bank of the Genetic Ichthyology Laboratory of the University of São Paulo. The FSHβ and LHβ subunit transcripts were repeatedly analyzed, using at least 3 different pituitaries per each region of the gene.

### Total RNA extraction

Total RNA was extracted from individual pituitary glands (wet weight: 70–100 mg) of *A*. *gigas* using the Purelink® total RNA Mini purification kit (Life Technologies, Carlsbad, CA, USA) and kept at the temperature of -70°C.

### Reverse-transcribed polymerase chain reaction (RT-PCR) and design of consensus or degenerated primers

All oligonucleotides used as primers were designed employing the Bio Edit® software [37]. Initially, nine (FSHβ) and six (LHβ) primer pairs were designed based on the conserved regions of 26 (FSHβ) and 25 (LHβ*)* fish sequences deposited in the GenBank, chosen among the species listed in Table 1, and these were used for different PCR test reactions. Complementary DNA (cDNA) was obtained from 0.25 μg of total pituitary RNA extracted from individual pituitary glands, using a GoScript® Reverse Transcription System (Promega, Madison, WIS, USA). After Oligo-dT and Random Primer addition, incubation was performed at 70°C for 5 min. GoScript® Reverse Transcriptase was then added before annealing at 25°C for 5 min, extending the reaction for 1 h at 42°C. Inactivation was carried out at 70°C for 15 min. PCR was performed in a Veriti 96-well Thermocycler (Applied Biosystems®, Foster City, CA, USA) with 0.2 μM (final concentration) of sense and antisense primers and 5.0 U of Taq DNA polymerase hf (Life Technologies). After an initial 2 min denaturation step at 94°C, thirty-five cycles were carried out: denaturation at 94°C for 30 s, annealing at 54°C (FSHβ) or 57°C (LHβ) for 30 s and extension at 68°C for 1 min. Final elongation was at 68°C for 5 min before holding at 4°C. In the case of FSHβ, one pair of consensus primers (#1 and #2, Table 2) provided 3 PCR fragments on 1.2% agarose gel, the least intense being confirmed to correspond to a putative partial FSHβ sequence, located approximately in the central region of the cDNA. In the case of LHβ, one pair of degenerate primers (#10 and #11) provided a single PCR fragment that corresponded to a putative partial LHβ sequence. Partial sequences of ag-FSHβ- and ag-LHβ-subunit cDNA were thus ready to use for the following reaction.

**Table 1:** Fish species and Genbank accession numbers for the sequences used in the amino acid percentage identity and phylogenetic analyses of the FSH and LH glycoprotein beta subunits.

Organism	LH		FSH		
	Nucleotide (mRNA)	Amino acid	Nucleotide (mRNA)	Amino acid	
*Arapaima gigas*	KJ741848	this paper	KJ729119	this paper	Osteoglossomorpha, Osteoglossiformes, Arapaimidae
*Acipenser baerii*	AJ251656	CAB93502	AJ251658	CAB93504	Chondrostei, Acipenseriformes, Acipenseridae
*Acipenser schrenckii*	AY575921	AAS92239	AY575920	AAS92238	Chondrostei, Acipenseriformes, Acipenseridae
*Acipenser sinensis*	EU523733	ACB29495	EU523732	ACB29494	Chondrostei, Acipenseriformes, Acipenseridae
*Anguilla anguilla*	X61039	CAA43374	AY148427	AAN64352	Elopomorpha, Anguilliformes, Anguillidae
*Anguilla japonica*	AB175835	BAD14302	AB016169	BAA36546	Elopomorpha, Anguilliformes, Anguillidae
*Anguilla marmorata*	FJ490347	ACK87153	FJ490346	ACK87152	Elopomorpha, Anguilliformes, Anguillidae
*Conger myriaster*	AB045158	BAB97391	AB045157	BAB97390	Elopomorpha, Anguilliformes, Congridae
*Misgurnus anguillicaudatus*	AB603807	BAK39639	AB603806	BAK39638	Ostariophysi, Cypriniformes, Cobitidae
*Carassius auratus*	D88024	BAA13531	D88023	BAA13530	Ostariophysi, Cypriniformes, Cyprinidae
*Ctenopharyngodon idella*	EF565171	ABQ51327	EF552359	ABQ42694	Ostariophysi, Cypriniformes, Cyprinidae
*Danio rerio*	AY424304	AAR84283	AY424303	AAR84282	Ostariophysi, Cypriniformes, Cyprinidae
*Hypophthalmichthys nobilis*	EF565164	ABQ42715	EF552360	ABQ42695	Ostariophysi, Cypriniformes, Cyprinidae
*Pimephales promelas*	DQ242617	ABB51645	DQ242616	ABB51644	Ostariophysi, Cypriniformes, Cyprinidae
*Clarias gariepinus*	X97761	CAA66359	AF324541	AAN75753	Ostariophysi, Siluriformes, Clariidae
*Ictalurus punctatus*	AF112192	AAG32156	AF112191	AAG32155	Ostariophysi, Siluriformes, Ictaluridae
*Silurus meridionalis*	AY973946	AAY42269	AY973947	AAY42270	Ostariophysi, Siluriformes, Siluridae
*Coregonus autumnalis*	L23431	AAA68207	L23432	AAA68208	Protacanthopterygii, Salmoniformes, Salmonidae,
*Brachymystax lenok*	AY515501	AAR99811	AY515500	AAR99810	Protacanthopterygii, Salmoniformes, Salmonidae
*Oncorhynchus mykiss*	AB050836	BAB17687	AB050835	BAB17686	Protacanthopterygii, Salmoniformes, Salmonidae
*Gadus morhua*	DQ402374	ABD62884	DQ402373	ABD62883	Paracanthopterygii, Gadiformes, Gadidae
*Odontesthes bonariensis*	AY319833	AAP85607	AY319832	AAP85606	Acanthopterygii, Atheriniformes, Atherinopsidae
*Fundulus heteroclitus*	M87015	AAB59963	M87014	AAB59962	Acanthopterygii, Cyprinodontiformes, Fundulidae
*Trichogaster trichopterus*	AF157631	AAD51935	AF157630	AAD51934	Acanthopterygii, Perciformes, Osphronemidae
*Trachurus japonicus*	KC787605	AGO59025	KC787604	AGO59024	Acanthopterygii, Perciformes, Carangidae
*Channa maculata*	AY447037	AAS01609	AY447038	AAS01610	Acanthopterygii, Perciformes, Channidae
*Haplochromis burtoni*	HQ147565	ADQ42414	HQ147566	ADQ42415	Acanthopterygii, Perciformes, Cichlidae
*Oreochromis niloticus*	AY294016	AAP49576	AY294015	AAP49575	Acanthopterygii, Perciformes, Cichlidae
*Pseudolabrus sieboldi*	AB300391	BAF81901	AB300390	BAF81900	Acanthopterygii, Perciformes, Labridae
*Amphiprion melanopus*	FJ868868	ACR08088	FJ868867	ACR08087	Acanthopterygii, Perciformes, Pomacentridae
*Dicentrarchus labrax*	AF543315	AAN40507	AF543314	AAN40506	Acanthopterygii, Perciformes, Moronidae
*Morone saxatilis*	L35096	AAC38019	L35070	AAC38035	Acanthopterygii, Perciformes, Moronidae
*Epinephelus coioides*	AF507939	AAM28896	AY553982	AAS60199	Acanthopterygii, Perciformes, Serranidae
*Acanthopagrus schlegelii*	EF605276	ABQ96864	AY921613	AAX18926	Acanthopterygii, Perciformes, Sparidae
*Pagrus major*	AB028213	BAB18564	AB028212	BAB18563	Acanthopterygii, Perciformes, Sparidae
*Scomber japonicus*	JF495133	AEN14605	JF495132	AEN14604	Acanthopterygii, Perciformes, Scombridae
*Thunnus thynnus*	EF205591	ABP04050	EF208026	ABP04057	Acanthopterygii, Perciformes, Scombridae
*Paralichthys olivaceus*	AB042423	BAB47388	AB042422	BAB47387	Acanthopterygii, Pleuronectiformes, Paralichthyidae
*Solea senegalensis*	EU100410	ABW81404	EF617342	ABU95601	Acanthopterygii, Pleuronectiformes, Soleidae
*Sebastes schlegelii*	AY609080	AAU14142	AY609079	AAU14141	Acanthopterygii, Scorpaeniformes, Sebastidae
*Monopterus albus*	EU840258	ACF70665	JN381164	AET99103	Acanthopterygii, Synbranchiformes, Synbranchidae

**Table 2:** Primers used in cloning *A*. *gigas* FSHβ and LHβ subunits cDNAs.

Number	Direction	Name	Sequence
Primer 1	Sense	FSHβ consensus-1	5’ ATG CAG CTG GTT GTC ATG GCA 3’
Primer 2	Antisense	FSHβ consensus-2	5’ TCT GGC CAC AGG GTA GGT GA 3’
Primer 3	Antisense	ag-FSHβ-1	5’ ACA GGG TAG GTG AAA T 3’
Primer 4	Antisense	ag-FSHβ-2	5’ GGG TCC ACT CCT TCA GGG 3’
Primer 5	Sense	AAP	5’ GGC CAC GCG TCG ACT AGT ACG GGI IGG GII GGG IIG 3’
Primer 6	Sense or antisense	AUAP	5’ GGC CAC GCG TCG ACT AGT AC 3’
Primer 7	Antisense	AP	5’ GGC CAC GCG TCG ACT AGT ACT TTT TTT TTT TTT TTT T 3’
Primer 8	Sense	ag-FSHβ-3	5’ CCC TGA AGG AGT GGA CCC 3’
Primer 9	Sense	ag-FSHβ-5’UTR	5’ GCT GGT AGG AGT CCA ACA G 3’
Primer 10	Sense	LHβ degenerate-1	5’ CTG GTG TTY CAR ACM WCC ATC T 3’
Primer 11	Antisense	LHβ degenerate-2	5’ AGT CMG ASG TGT CCA TKG TG 3’
Primer 12	Sense	ag-LHβ-1	5’ GGG CGT GAG GTA CGA GA 3’
Primer 13	Antisense	ag-LHβ-2	5’ CAT TGA GGG AAC AAA ACT 3’
Primer 14	Antisense	ag-LHβ-3	5’ TGA TAT TTA GGG TTC GGG TTA GTT C 3’
Primer 15	Sense	ag-LHβ-5’UTR	5’ TAT CTC GGC TGC CGC TTG TT 3’

### Rapid amplification of cDNA ends (RACE) for FSHβ and LHβ sequencing

For 5’-RACE and 3’-RACE, 0.5 μg of total RNA were reverse-transcribed with 200 U of Superscript II (Life Technologies), either for FSHβ or LHβ sequencing.

In the case of FSHβ, gene-specific primer #3 was used for 5’-RACE, according to kit instructions, to get a single-stranded cDNA. The Abridged Anchor Primer (#5) was then used together with gene-specific primer #4 to obtain a PCR fragment that was finally purified and re-amplified using specific primer #4 and the Abridged Universal Amplification Primer (#6) from the kit. This finally provided a 356 bp sequence corresponding to the 5’ region of FSHβ cDNA. The Adapted Primer (#7) was used for 3’RACE for obtaining single-stranded cDNA, according to kit instructions. The gene-specific primer #8 was then used together with the Abridged Universal Amplification Primer (#6). A single intense PCR fragment was obtained, which provided a 370 bp correct sequence corresponding to the 3’ region of FSHβ cDNA. To confirm the whole FSHβ cDNA sequence, gene-specific primer #9, designed right at the beginning of the 5’-UTR sequence was used together with the Abridged Universal Amplification Primer.

In the case of LHβ, the 3’-RACE strategy was applied first, since, via degenerate primers, a sequence apparently closer to the 3’ region had been obtained in the first phase. Adapted Primer (#7) was used for obtaining single-stranded cDNA. Gene-specific primer #12, together with the Abridged Universal Amplification Primer (#6), gave a single intense PCR fragment that, after sequencing, provided a 409 bp sequence corresponding to the 3’ region of LHβ cDNA. Gene-specific primer #13 was then used for 5’-RACE, obtaining single stranded cDNA. Abridged Anchor Primer (#5), together with gene-specific primer #14, gave a single, intense PCR fragment that provided a 624 bp sequence corresponding to the 5’ region of LHβ cDNA. To confirm the whole LHβ cDNA sequence, gene-specific primer #15, designed right at the beginning of the 5’-UTR sequence was used together with the Abridged Universal Amplification Primer (#6).

The complete amino acid sequences of ag-FSHβ and of ag-LHβ subunits were determined and putative signal peptides were predicted using the “Signal peptide 4.1 Software” [38].

### Molecular modeling

The initial three dimensional (3D) models of ag-GTHα, ag-FSHβ and ag-LHβ were generated by employing the threading modeling method [39,40], carried out using the HHpred server [41] and Modeller Software [42–44]. Initially, the HHpred server generated 1D alignments for the GTHα, FSHβ and LHβ subunits with sequences of the three dimensional structures of the proteins available at the Protein DataBank (PDB), employing a global alignment method and matching the predicted secondary structure of the models. The sequence of ag-GTHα, from Faria et al. [36], and those of ag-FSHβ and ag-LHβ, obtained in the present work, were aligned with human FSH and human chorionic gonadotropin (hCG) available at the PDB under IDs 4ay9, chains a and b [45,46], and 1hcn, chain b [47], respectively. All templates showed 100% of probability and identity values >47% when compared to their respective models (S1 Table).

After the primary alignments of the 3 subunits, initial 3D models were constructed using the Modeller Software. The 5 lowest energy models were chosen from the 60 that were generated and employed to obtain the optimum model for Molecular Dynamics simulations by evaluating their stereochemical quality via Ramachandran plots [48]. Both heterodimers were constructed by superimposing each modeled subunit on its respective crystallographic template (4ay9 and 1hcn).

#### Molecular Dynamics simulation

After the construction of the initial FSH and LH dimer models, the Molecular Dynamics (MD) simulation of these two modeled hormones was performed. All the MD parameters were equal for the two models generated, the simulation being carried out by employing the GROMACS v.4.5.4 software [49, 50] and using an explicit solvation system in which water molecules were represented by a Simple Point Charge model. The protonation states of the charged groups were those at pH 7.0 and counterions were added to insure electrical neutrality of the system at 0.1M NaCl. The Gromos Force Field [51] was chosen for the MD simulations at constant temperature and pressure in a periodic truncated cubic box in which the minimum distance between any atom of the protein and the box wall was 2.0 nm. Initially, an energy minimization based on the steepest descent algorithm was performed; subsequently, 20 ps of MD simulation, fixing the backbone atom positions, were applied at a temperature of 310°K to relax the system. Finally, an unrestrained MD simulation was performed for 20 ns at 310°K to assess the stability of the structures. During all simulations, the temperature and pressure (1.0 bar) were maintained constant [52].

#### Structural analysis and validation

The final model, generated by the MD simulations, was checked using several GROMACS structural analyses, as well as Ramachandran plots generated with the Rampage server [48]. The pseudo-energy profile of the models was analyzed via the Verify 3D server [53] and ProSA-web [54]. It is also important to emphasize that all protocols performed were based on current literature [55–57].

### Phylogenetic analysis

The phylogenetic analysis was carried out on the basis of MUSCLE alignments [58] of the mature peptide DNA sequences of the FSHβ and LHβ subunits of 41 fishes, including *A*. *gigas*. The species and sequences used are presented in Table 1 and were obtained from the GenBank/EMBL. The alignment and identity calculations between the pairwise sequences were carried out via Geneious 5.5.6 and the search for the best substitution model and the phylogenetic trees via MEGA 6 [59]. All positions containing gaps and missing data were not considered in the analysis. The DNA sequence alignments from FSHβ and LHβ were concatenated to generate trees based on the methods of Maximum Parsimony (MP) and Maximum Likelihood (ML), applying the HKY model [60] with Gamma parameter = 1.34. The bootstrap test [61] with 1,000 replicates was conducted for all analyses, and the three Chondrostei (*Acipenser* species) were set as outgroup. Branches with less than 50 percent of the bootstrap replicates were collapsed.

### Synthesis of ag-FSH in human embryonic kidney 293 (HEK293) cells

The transient transfection and synthesis of ag-FSH was carried out using the Expi293^TM^ Expression System Kit (Life Technologies, Carlsbad, CA, USA), following the manufacturer’s instructions. Briefly, each ag-FSH α- and β-subunit cDNA was separately inserted into a pcDNA^TM^ 3.4-TOPO^®^ vector, adding then 30 μg of plasmid DNA (15 μg for each subunit vector) to ExpiFectamine^TM^ 293 Reagent, in Opti-MEM^®^ I medium, incubating for 20–30 min at room temperature to obtain the DNA-ExpiFectamine^TM^ 293 Reagent complex. The mixture (3 ml) was finally added to a 125-ml Erlenmayer shaker flask containing 7.5 x 10^7^ Expi293F^TM^ cells in Expi293^TM^ Expression Medium, to a final volume of 28.5 ml. A negative control flask was also prepared by adding 3 ml of Opti-MEM® I medium instead of the DNA-ExpiFectamine^TM^ Reagent complex. Transfection was thus carried out in a 37°C incubator with a humidified atmosphere of 8% CO_2_ in air, in an orbital shaker rotating at 125 rpm. Approximately 16–18 hours post-transfection, Transfection Enhancer 1 and Transfection Enhancer 2 were added, thus reaching a final volume of 30 ml in each 125-ml flask. Media, containing the secreted recombinant protein of interest, were harvested beginning at approximately 48 hours post-transfection and assayed for recombinant protein expression.

### Conditioned medium purification by reversed-phase HPLC (RP-HPLC)

The first purification step was carried out via RP-HPLC on a Vydac C4 column (300 Å pore size, 5 μm particle size, 25 cm x 4.6 mm I.D) from Grace-Vydac (Fisher Scientific, USA) by applying 5 ml of transfection conditioned medium. Elution and gradient conditions were those described in Loureiro et al. [62], that were specifically set up for running undissociated FSH heterodimer. Under the same conditions, 5 ml of the negative control of transfection were also chromatographed on the same RP-HPLC column.

### Ag-FSH purification by high-performance size exclusion liquid chromatography (HPSEC)

The pool of ag-FSH-derived material, which was evidently not present in the negative control, was applied to a HPSEC G2000 SW column (60 cm x 7.5 mm I.D., particle size of 10 μm and pore size of 125 Å), purchased from Tosoh Bioscience (Tokyo, Japan), eluting as described [63]. A similar pool, obtained from the RP-HPLC negative control, was applied to the same HPSEC column. A reference preparation, human FSH-SIAFP-B-3 (lot # AFP7298A), from the National Hormone & Peptide Program (NHPP, Torrance, CA, USA), was also applied.

### Ag-FSH dissociation into α- and β-subunits

For heterodimer dissociation of ag-FSH into its subunits we utilized a methodology previously set up for pituitary or recombinant human glycoprotein hormones (5–10 μg in 50–100 μl phosphate-buffered saline), incubating overnight at 37°C in 5M acetic acid [64].

## Results

### cDNA sequencing

The DNA sequences obtained via 3’ and 5’-RACE using material from at least three different pituitary glands were compared and aligned. Overlapping enabled their proper joining into nucleotide sequences spanning the entire cDNA.

**Fig 1:**
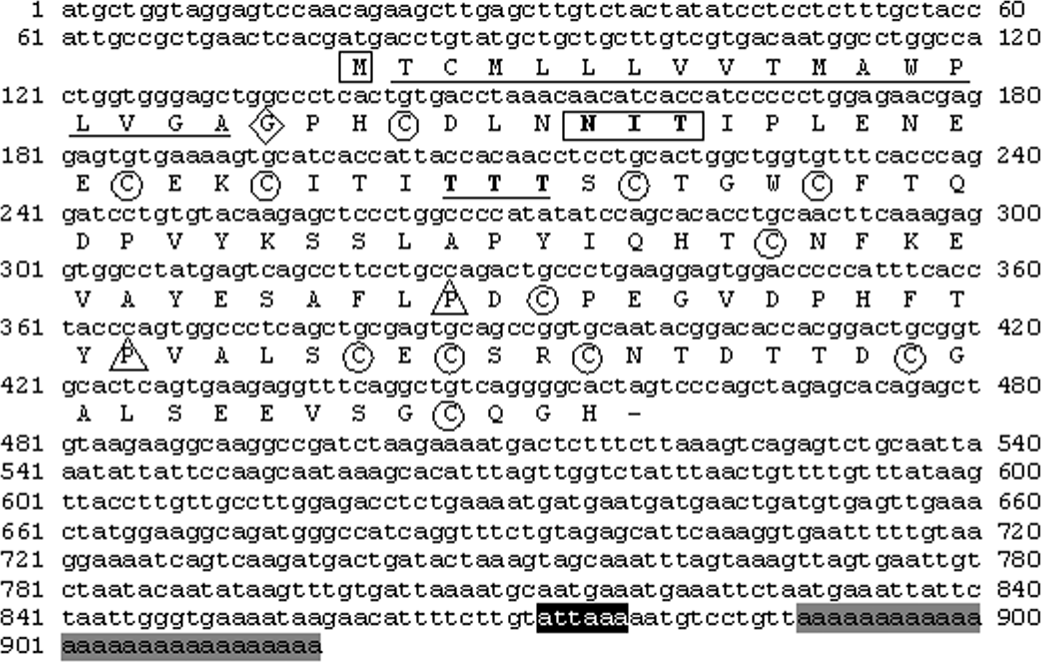
Nucleotide and deduced amino acid sequence of the cDNA encoding for the FSHβ-subunit of *A*. *gigas*. M, inside a square, start coding region; G, inside a rhombus, first amino acid of the mature peptide; N I T, inside a rectangle, glycosylation site; C, inside a circle, conserved cysteine residues; P, inside a triangle, conserved proline residues; attaaa, designated with a black square, polyadenylation signal; aaaaaaaaaaaa, designated with a gray square, poly(A+) tail. GenBank Accession number: KJ729119.

**Fig 2:**
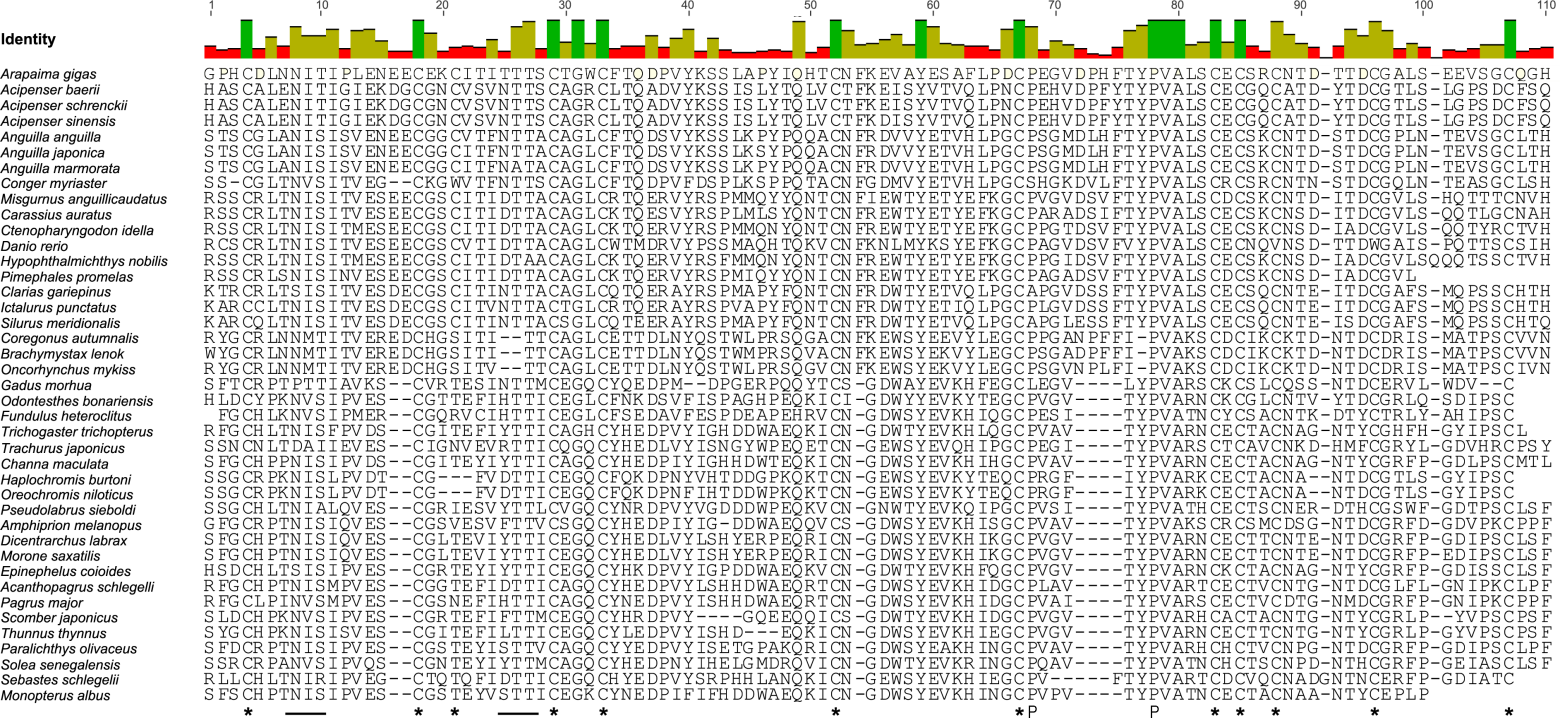
Alignment of the FSHβ mature peptides of three Chondrostei and 38 teleosts, including *A*. *gigas*. The Genbank accession numbers for these sequences are given in Table 1. Below the alignment the conserved cysteines(*), prolines (P) and the putative N-linked glycosylation sites (___) are highlighted.

The *A*. *gigas FSHβ* cDNA sequence (Fig 1) was 913 bp in total length and had an open reading frame of 381 bp beginning with the first ATG codon at position 79 bp (78 bp 5’-UTR) and ending with the stop codon at position 457 bp, presenting a 434 bp 3’-UTR. A polyadenylation signal (ATTAAA) was recognized 13 bp upstream from the poly (A^+^) tail. The coding region translates into a polypeptide of 126 amino acids, while the cleavage site for the putative signal peptide was predicted to be between amino acids 18 and 19. This provides a mature peptide of 108 and a signal peptide of 18 amino acids. The proposed mature peptide of ag-FSHβ thus contains 12 conserved cysteines and 2 conserved prolines (Fig 1). A putative N-linked glycosylation site, the only one predicted, was identified at amino acid position (AA) 8–10 (NIT), while a second possible N-linked glycosylation site was lost at AA 25–27 (TTT), as can be seen in the alignment of the mature peptide of ag-FSHβ with the other 37 teleosts and 3 Acipenseriformes (Fig 2).

**Fig 3:**
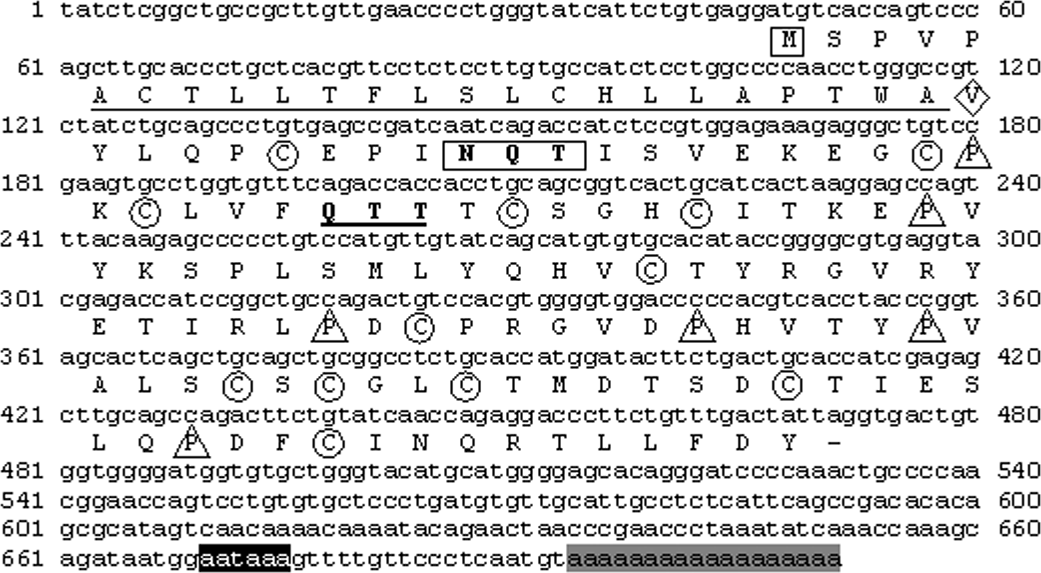
Nucleotide and deduced amino acid sequence of the cDNA encoding for the LHβ-subunit of *A*. *gigas*. M, inside a square, start coding region; V, inside a rhombus, first amino acid of the mature peptide; N Q T, inside a rectangle, glycosylation site; C, inside a circle, cysteine residues; P, inside a triangle, proline residues; aataaa, designated with a black square, polyadenylation signal; aaaaaaaaaaaa, designated with a gray square, poly(A+) tail. GenBank Accession number: KJ741848.

**Fig 4:**
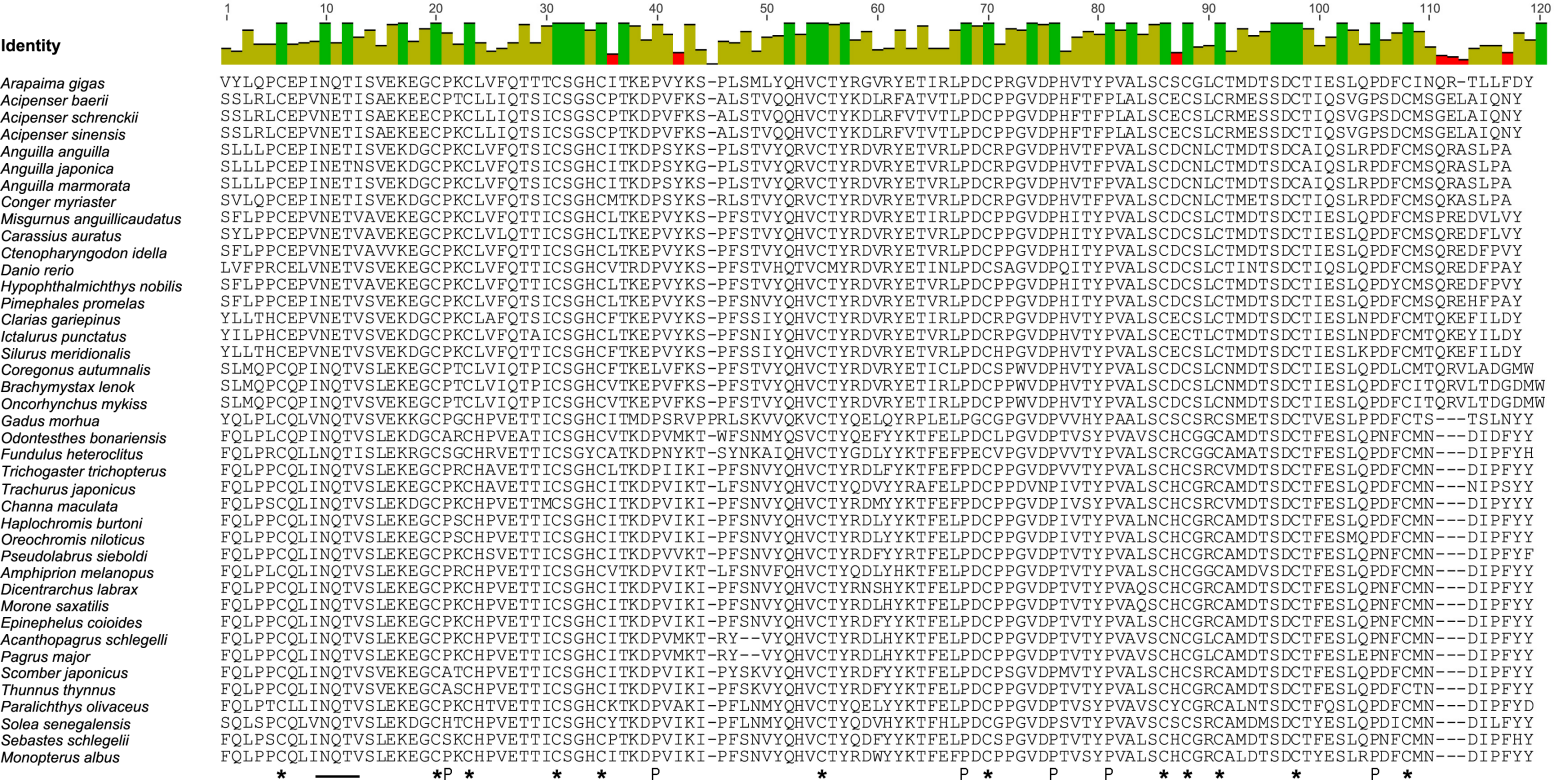
Alignment of the LHβ mature peptides of three Chondrostei and 38 teleosts, including *A*. *gigas*. The Genbank accession numbers for these sequences are given in Table 1. Below the alignment the conserved cysteines (*), prolines (P) and the putative N-linked glycosylation site (___) are highlighted.

The *A*. *gigas* LHβ cDNA sequence (Fig 3) was 711 bp in total length and had an open reading frame of 426 bp beginning with the first ATG codon at position 47 bp (46 bp 5’-UTR) and with the stop codon at position 469, presenting a 221 bp 3’-UTR. A polyadenylation signal (AATAAA) was recognized 18 bp upstream from the poly (A^+^) tail. It is interesting to observe that the non-consensus polyadenylation signal ATTAAA, already found in ag-GTHα cDNA [36] and here confirmed in ag-FSHβ, was replaced in ag-LHβ cDNA by the very highly conserved AATAAA [65]. The coding region translates into a peptide of 141 amino acids, while the cleavage site for the putative signal peptide was predicted to be between amino acid 24 and 25. This provides a mature peptide of 117 and a signal peptide of 24 amino acids. The proposed mature peptide of ag-LHβ thus contains 12 conserved cysteines and 6 conserved prolines (Fig 3). A putative N-linked glycosylation site was identified at AA 10–12 (NQT), while a second possible N-linked glycosylation site was lost at AA 27–29 (QTT) of the mature peptide (Fig 4).

The comparison between the amino acid sequences of FSH and LH β-subunits of *A*. *gigas* reveals an identity of 49.5%. Table 3 presents the percent identities of LHβ and FSHβ mature peptides for 13 fish orders. The percent identities of the ag-FSHβ and ag-LHβ mature peptides, in comparison with the corresponding human sequences, were 45.1% and 51.4%, respectively.

**Table 3:** Percentage identity of FSHβ and LHβ peptides among fish orders (FSHβ above the diagonal).

			1	2	3	4	5	6	7	8	9	10	11	12	13
1	Teleostei Osteoglossomorpha	Osteoglossiformes (*A*. *gigas*)	-	51.1	60.9	51.6	53.0	44.1	33.0	37.6	37.4	36.3	35.7	36.4	35.0
2	Chondrostei	Acipenseriformes (3)	55.6	-	48.7	44.7	49.4	39.0	27.8	35.2	33.9	33.4	32.0	32.4	33.3
3	Teleostei Elopomorpha	Anguilliformes (4)	74.3	64.7	-	54.4	56.6	43.8	33.2	41.0	40.6	39.0	39.8	36.1	39.7
4	Teleostei Ostaryophysi	Cypriniformes (6)	75.6	63.4	77.8	-	66.6	47.0	33.6	40.0	37.9	39.7	40.4	40.8	39.1
5	Teleostei Ostaryophysi	Siluriformes (3)	74.9	61.3	75.7	80.2	-	44.8	35.5	40.4	38.0	40.5	41.3	37.9	39.8
6	Teleostei Protacanthopterygii	Salmoniformes (3)	74.2	59.3	71.7	76.4	73.1	-	32.1	36.7	36.4	36.7	36.7	39.1	31.7
7	Teleostei Paracanthopterygii	Gadiformes (1)	53.8	48.7	52.1	53.3	54.9	49.7	-	49.0	41.0	48.5	49.1	45.2	49.0
8	Teleostei Acanthopterygii	Atheriniformes (1)	57.6	46.6	53.0	56.6	58.5	57.1	59.8	-	60.0	58.1	57.3	51.9	59.4
9	Teleostei Acanthopterygii	Cyprinodontiformes (1)	55.1	47.5	52.1	54.8	54.8	51.6	59.0	70.7	-	53.9	49.5	49.0	58.5
10	Teleostei Acanthopterygii	Perciformes (14)	65.1	52.4	59.7	65.5	63.0	63.5	63.5	80.5	74.7	-	63.0	56.0	66.4
11	Teleostei Acanthopterygii	Pleuronectiformes (2)	59.7	50.4	55.4	61.3	60.7	59.1	60.2	76.7	69.0	79.5	-	56.7	66.6
12	Teleostei Acanthopterygii	Scorpaeniformes (1)	62.7	51.4	57.9	63.3	61.9	61.9	61.5	83.6	75.0	88.9	80.6	-	59.2
13	Teleostei Acanthopterygii	Synbranchiformes (1)	64.4	50.5	60.2	64.7	62.7	62.2	62.4	79.3	73.3	89.5	81.0	88.8	-

### Molecular modeling

**Fig 5:**
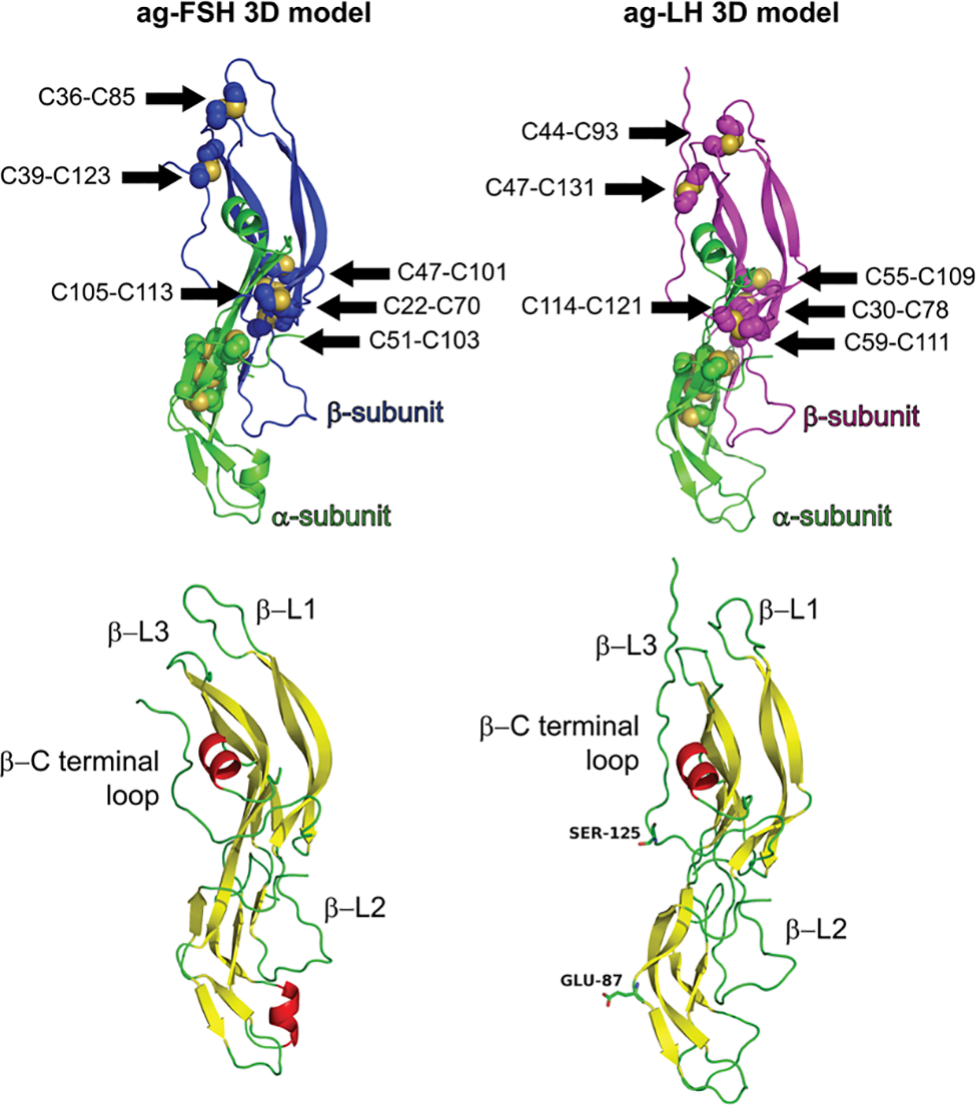
Tridimensional models obtained after molecular dynamics simulations. On the top, the ag-FSH and ag-LH structures highlighting the α-subunit (green cartoons) and the β-subunit (blue for FSH and magenta for LH) and the predicted dissulfide bonds of the β-subunits are shown. On the bottom, the 3D models are presented in cartoon style and colored according to the secondary structure (helices in red, sheets in yellow and loops in green), highlighting the predicted loops of the β-subunits and the residues predicted in the outlier regions of the Ramachandran plot.

**Fig 6:**
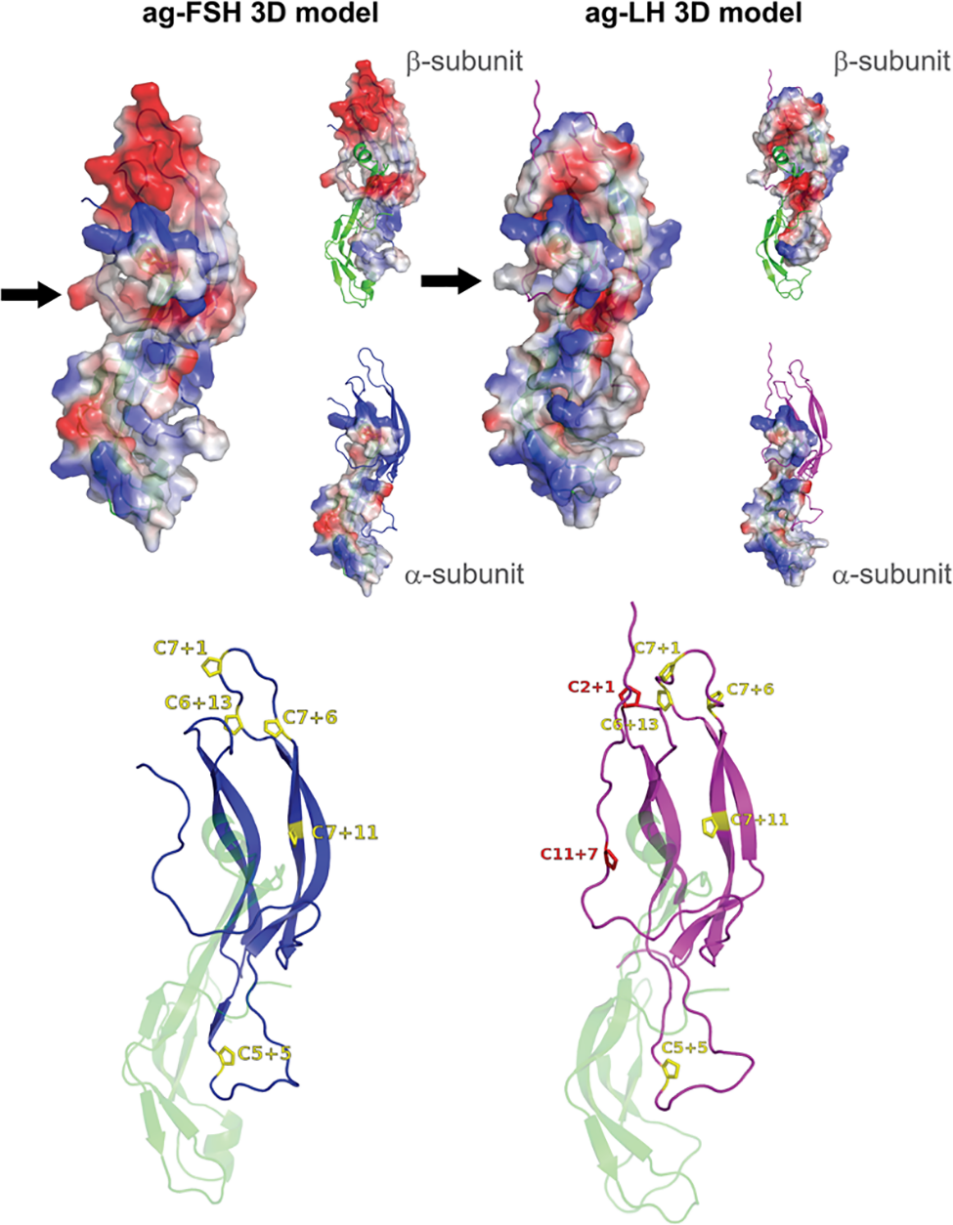
On the top, electrostatic potential surface of ag-FSH (left) and ag-LH (right) and surface of α- and β-subunits. The surface is colored in blue (positively charged groups) and red (negatively charged groups), while neutral groups are in white. The black arrow indicates the “seat-belt” structure of the β-subunits. On the bottom, localization of 5 (ag-FSH) and 7 (ag-LH) conserved proline residues in the hormone structures. Each proline is identified according to its position in relation to the previous cysteine residue.

Fig 5 displays in the upper part the average structure in solution of ag-FSH and ag-LH, highlighting the two subunits, the predicted disulfide bridges and the loops. The so-called “seat belt” in the β subunits, known to wrap around the α subunit, can also be identified between the 3^rd^ and 12^th^ cysteine: C39 and C123, for ag-FSH, and C47 and C131, for ag-LH. In the lower part of Fig 5, the 3D models are presented in cartoon style, highlighting the predicted secondary structures (helices and sheets) and the two residues located in the outlier regions of Ramachandran plots. In Fig 6 (top) the electrostatic potential surfaces of ag-FSH and ag-LH and the partial surfaces of the α- and β-subunits are shown. In Fig 6 (bottom) the proline residues, which were found to be conserved by comparing the four human and *A*. *gigas* gonadotropin sequences, are located in the β-sheet/loop regions.

Several structural parameters, such as the root-mean-squared deviation (RMSD) of the backbone atom positions indicating structural stability, the radius of gyration indicating protein compaction, the hydrophobic and hydrophilic surface areas indicating solvent exposure and the number of hydrogen bonds between protein atoms (S1 Fig), confirmed that the structures of both hormones had stabilized by around 15 ns of MD simulation. The last 5 ns of simulation were thus analyzed. It is important to observe that, during the 20 ns simulation, physicochemical parameters such as the total energy, periodic box volume and pressure, temperature and density of the system were stable, showing only very small variations: this indicates the stability of the simulated system (S2 Table).

Both 3D models were validated with ProSA-web, all residues being found under 0 score, and with “Verify 3D”, in which only the C-terminus of ag-LHβ was above 0 score; according to the Ramachandran plot, ag-LH presented only 2 residues that were outliers in the loop region (S3 Table).

The calculated root-mean-square fluctuations (RMSF) of both hormones are shown in S2 Fig. As expected, the RMSF for the α subunits suggests that their backbones have almost identical profiles. In the case of the β subunits, minor differences in the RMSF values are observed, also suggesting similar backbone movements.

A comparison between the seat-belt amino acid composition of ag-LH, ag-FSH and all gonadotropins analyzed by Aizen et al. [66] is shown in S3 Fig.

### Phylogenetic analysis

The combined analysis of FSHβ and LHβ DNA sequences presented in S4 Fig shows that most branches have high bootstrap support, except within the Acanthomorpha superorder, which is in fact collapsed in this figure. Although not shown in the figure, supports above 50% were found in Pleuronectiformes (above 80%) and in the Perciformes clades: Cichlidae (100%), Scombridae (above 95%), Moronidae (100%), Sparidae (100%), Moronidae+Sparidae (above 80%) and in the group formed by *Channa maculata* (Channidae) and *Trichogaster trichopterus* (Osphronemidae) (71.8% for ML and 60.2% for MP). Maximum parsimony also supports a clade formed by *Pseudolabrus sieboldi* (Labroidei, Labridae) and *Epinephelus coioides* (Percoidei, Serranidae) (76.3%) within Acanthomorpha. The overall phylogenetic relationships within the Acanthomorpha, however, could not be fully resolved.

### Ag-FSH synthesis in HEK293 cells

**Fig 7:**
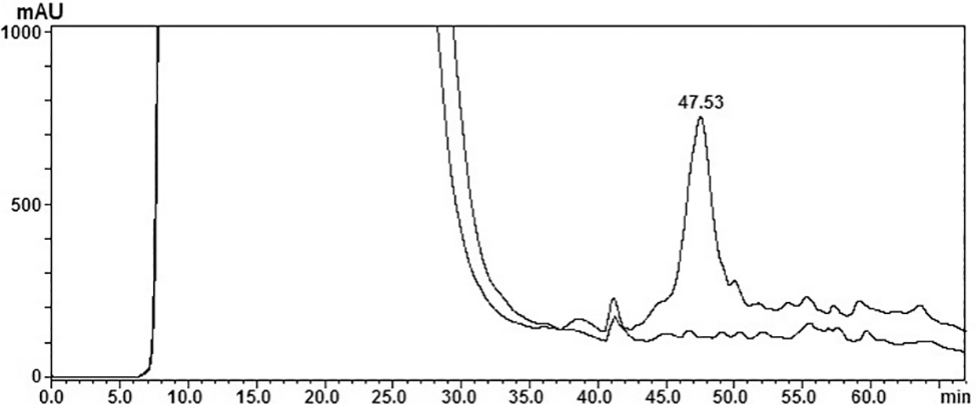
First purification step of HEK293F conditioned medium via RP-HPLC. The chromatogram is derived from the application of 5 ml of ag-FSH transfection conditioned medium, in comparison with an analogous chromatogram derived from the application of 5 ml of the negative control of transfection.

**Fig 8:**
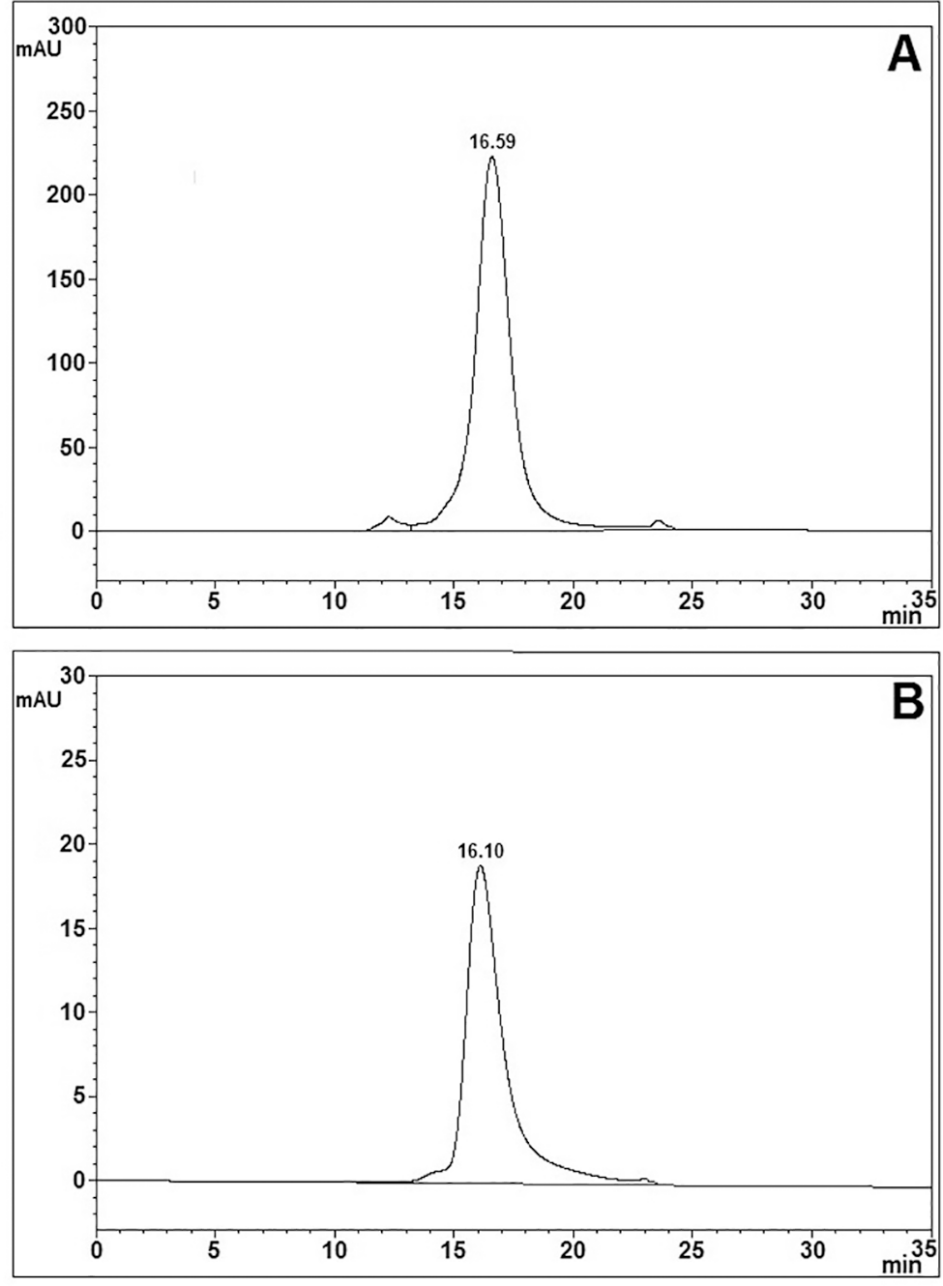
**HPSEC obtained upon application of: (A) 0.25 ml of a pool of ag-FSH-derived material from RP-HPLC (see Fig 7) and already purified once on the same HPSEC; (B) 5μg/5μl of human pituitary FSH reference preparation from NHPP, run under identical conditions**.

**Fig 9:**
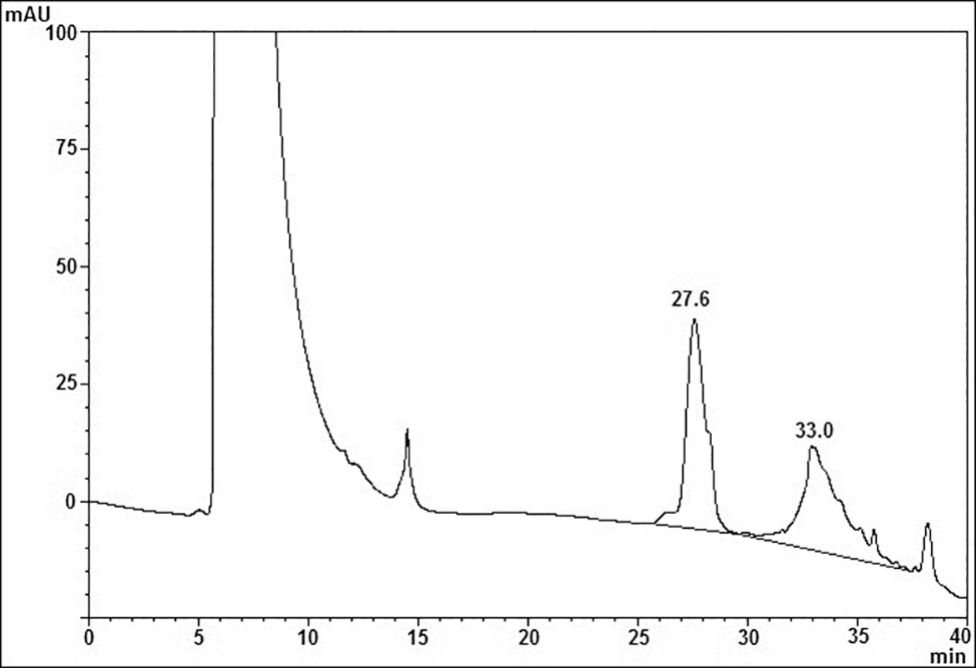
RP-HPLC, run under dissociating conditions as described [64], after application of a sample of ag-FSH obtained from HPSEC (see Fig 8) and incubated overnight with 5M acetic acid at 37°C.

As shown in Fig 7, using a particular RP-HPLC condition that does not dissociate FSH heterodimer [62], ag-FSH-related material was obtained from HEK293 conditioned medium, while no material appeared in the same position when the negative control of transfection was run under the same conditions. A pool of this ag-FSH-related material was then purified twice on HPSEC, presenting a symmetrical peak whose t_R_ is quite close to that shown by the human FSH reference preparation, run under the same conditions (Fig 8). A complete dissociation of ag-FSH into its α- and β-subunit, after overnight incubation with 5M acetic acid, following an analogous protocol that we have previously described for hFSH subunit dissociation [64], provided a very similar pattern (Fig 9). A preliminary quantification concerning ag-FSH expression in this HEK293 cell line, adapted to high density, serum-free suspension culture, indicated a yield of ~28 mg/liter.

## Discussion

The mature peptides of the two beta subunits (FSH and LH) are derived from the same ancestral gene [67,68] and the comparison between the amino acid sequences of the FSHβ and LHβ subunits of *A*. *gigas* reveals an identity of 49.5%, which is higher than that calculated for other fishes, ranging from 32 to 40% [19,23,69]. Besides the 12 conserved cysteines and the first N-glycosylation site (NxT), the other interesting conserved positions of FSHβ and LHβ in fishes are related to the second putative N-linked site (absent in both *A*. *gigas* beta subunits), to a conserved region between Cys7 and Cys8 and to the dimer interface positions, similar to what has been reported for other glycoprotein hormone beta chains [45,70]. In the position related to the second putative N-linked glycosite, both human forms, for example, present an active NTT site, while ag-FSHβ presents a TTT sequence and ag-LHβ a QTT sequence. As far as we know, the conserved region between the 7^th^ and 8^th^ Cys (relative to tetrapods) does not seem to be related to any known function. Interestingly, this region concentrates conserved proline residues that can be essential for the protein structure of gonadotropin beta subunits, since prolines tend to bend the regional amino acid alignment and therefore to fold the protein [71,72].

The main characteristic of the primary structure of the FSH and LH beta subunits is the presence of 12 conserved cysteines. These are important for determining the cysteine-knot structure comprising 3 disulfide bridges [14] for stabilizing the heterodimer [73] and for hormone activity [74–76]. The alignment of the mature peptides shows that this structure is highly conserved in fish LHβ subunits but not in FSHβ, which is less conserved both in structure and sequence [1]. The table of the percentage of amino acid identities clearly shows that FSHβ is more variable than LHβ (27.8–66.6% and 46.6–89.5%, respectively), which contrasts with Tetrapoda, where LHβ diverged faster than FSHβ. According to So et al. [23], the significantly lower conservation in fish FSHβ implies a functional divergence of FSH, and raises an interesting question about the roles and physiological relevance of FSH in different groups of teleosts. It is noteworthy, moreover, that ag-GTHα [36], ag-FSHβ and ag-LHβ show 67%, 45% and 51%, respectively, sequence identity with the corresponding human mature peptides and 66%, 67% and 63% with rat mature peptides. These identity values, of the same order as those existing between the human and rat sequences (72%, 65% and 63%), point, in our opinion, to a possible *in vivo* bioactivity detection for ag-FSH and ag-LH if analyzed in the classical female and male rat assay, normally carried out for human gonadotrophic hormone potency determination [77,78]. This is, however, only a hypothesis that needs to be experimentally verified with the purified recombinant hormones.

In our FSHβ alignment considering 41 species (Fig 2), only 14 positions are totally identical, 9 of which being cysteines. Two out of the three remaining conserved cysteines (the 10^th^ and 11^th^) can be found, however, in 40 of the fish sequences analyzed here, except in *Danio rerio*. The last non-conserved cysteine (the 3^rd^) is equivalent to the third tetrapod cysteine and, according to several authors, is present in ancient fish such as elasmobranchs [79], chondrosteans [80], eels [69] and Ostariophysi (Siluriformes and Cypriniformes), but not in Salmoniformes and Acanthomorpha (Acanthopterygii and Paracanthopterygii). The present alignment thus confirms these reports, since A. gigas, an ancient fish, also presents the 3^rd^ cysteine as in tetrapods.

Particular cases of FSHβ are *Clarias gariepinus* (Siluriformes), which lost the first N-glycosylation site, and the acanthopterygians species (*Epinephelus coioides*, *Sebastes schlegeli*, *Pseudolabrus sieboldi*, *Trachurus japonicus*), which lack any conserved N-linked sites. Additionally, unlike the other euteleosts, *Gadus morhua* lost the first but not the second putative N-glycosylation site.

Glycosylation is the most abundant post-translational modification of proteins and its impact may range from very subtle to crucial, since glycoproteins are involved in various biological roles. Based on the linkage between the amino acid and the sugar, glycosylation is classified into five types in eukaryotes, the most common being the N- and O-linked glycosylations [81]. Until now, only N-linked glycosylation was described for FSHβ and LHβ and these sites are highly conserved through Osteichthyes evolution, indicating their importance for these proteins; some fish species lack, however, this type of glycosite. N-linked glycosylations present the well-established consensus sequence N-X-S/T (X been any amino acid except Pro) [82,83], while the O-linked glycosylation, which occurs on the OH groups of Serine (S) or Threonine (T), is not known to occur in any consensus sequence in eukaryotes so far, which makes its prediction more difficult.

When comparing the *A*. *gigas* FSHβ and LHβ peptide sequences with those of other fishes, the lowest identities were found with Acanthomorpha orders (especially Gadiformes) and the highest identities with the basal teleosts Anguilliformes and Ostariophysi (Cypriniformes and Siluriformes). Although at a higher level of conservation, the same pattern was found by Faria et al. [36] comparing the ag-GTHα peptide with other fish orders, varying from 55 to 70% for Acanthomorpha and from 88.1 to 89.5% for Anguilliformes and Ostariophysi. In addition, the comparisons of ag-GTHα with Acipenseriformes (Chondrostei) and Salmoniformes also showed high identity values (87.1% and 75.7%, respectively). This also happens for FSHβ, while LHβ identity between *A*. *gigas* and the Acipenseriformes is lower than that found for most Acanthomorpha orders. Faria et al. [36] also considered that a relatively well-conserved alpha subunit present in the basal orders of Teleostei and Acipenseriformes gradually evolved to the GTHα forms present in Acanthomorpha.

As for other gonadotrophic hormones, the formation of a “seat-belt” in the β subunit structure that wraps around the α subunit, favored by the disulfide bond existing between an N-terminus (C3) and a C-terminus (C12) cysteine, is also observed in *A*. *gigas* 3D models [14,45,81]. Chatterjee et al. [84], however, noted that the 3^rd^ conserved cysteine is absent in snakehead fish (*Channa maculata*) FSH and, on the basis of their modeling, suggested that, thanks to the flexibility of the C-terminal loop, a disulfide bond could be formed between a positionally shifted N-terminal cysteine and the 12^th^ one. In our case it was observed that the electrostatic complementarity of both the α and β subunits in ag-FSH and ag-LH 3D models favors the seat belt formation. Residues included in this seat-belt region (between C10 and C12) have also been shown to be involved in ligand selectivity and in determining receptor specificity. The net charge difference in the region between the 10^th^ and the 11^th^ cysteine residue of the human gonadotropin β-subunit seems, in fact, to have selected FSH receptor-activation from LH receptor-activation [1].

While several studies have performed molecular modeling for human and for other mammalian GTHs, as far as we know only three works reported such models for fish species [66,84,85]. Particularly interesting is the study carried out by Aizen et al. [66] who analyzed pharmacologically and structurally (3D modeling) the interactions between GTHs and GTHRs of less evolved (eel and trout) and relatively modern (tilapia) fish. The authors highlighted the importance, in receptor activation, of a few residues located outside the hormone-receptor interface region determining diverse electrostatic and steric features in the different species. In the present work, dealing only with ligand and not with receptor models, a difference between the charge distribution in the upper region of the β-subunit of ag-FSH (negatively charged) and that of ag-LH (positively charged) versus a predominantly positive α-subunit is observed. The seat-belt region of both ag-FSHβ and ag-LHβ models displays similar electrostatic features that allow the α-subunit to bind the β-subunit. This equivalent heterodimer formation (and also the similar conformation and electrostatic profile of both α-subunits) is not in contrast with the “negative specificity” model described by Combarnous [86], which states that the common α-subunit could be responsible for receptor high-affinity binding, while the β-subunit for its specificity, determined by inhibiting sites that impede the binding of each dimer to the receptors of the other hormones. It can be suggestive to consider that in our ag-models the top surface of each β-subunit has a different electrostatic distribution. It would be necessary, however, to construct hormone-receptor structural models for ag-GTHs, in order to evaluate whether this change of the electrostatic map in the heterodimer could influence cross-type hormone specificities or cross-species activities.

Particularly interesting in the work of Aizen et al. [66] is the cross-species activation of eel-LHR by tr-LH, which could be explained by the very similar seat-belt sequences of eel-LH and tr-LH (14 identical and 3 similar out of 18 residues), the closest among all GTHs used in the study. As one can observe by adding ag-GTH sequences to the alignment carried out by these authors (S3 Fig), an even higher similarity is found between ag-LH and eel-LH (15 identical and 1 similar residue), or between ag-LH and ta-LH (15 identical and 3 similar), up to the amazing “quasi-identity” found between ag-LH and tr-LH: 17 identical and 1 similar, out of a total of 18 residues. On this basis, therefore, a significant pharmacological activation of eel-LHR, tr-LHR or ta-LHR by ag-LH would be expected. As far as we know, ag-GTHR sequences, which could provide additional hormone-receptor structural models, possibly predicting other cross-species ligand-receptor activations, are not yet available. Finally, still comparing the 3D models of ag-LH and ag-FSH with the human FSH models constructed by Aizen et al. [66], it is worth noting that the calculated fold of *A*. *gigas* gonadotropin models is very close to the reported human, bovine, porcine and fish fold. These data point to the stability of our proposed models and indicate that the calculated folds corroborate experimental and literature data [48, 50, 66].

It should be noted that the seven proline residues shown in Fig 7, which are totally (C6+13 and C7+11) or partially (C2+1, C5+5, C7+1, C7+6 and C11+7) conserved between the human and *A*.*gigas* FSH and LH structures, satisfy the predicted secondary structures of the β-subunits of the glycoprotein hormone family reported by Wako and Ishii [85] in their schematic representation. These proline residues are practically all located in β-sheet/loop inversion regions, confirming the already mentioned tendency of prolines to bend the backbone direction and thus participate in protein folding [72, 87, 88].

The resulting phylogenetic tree of the concatenated FSHβ and LHβ sequences of mature peptide cDNAs placed *A*. *gigas* (Osteoglossomorpha) as the sister group of Clupeocephala (Ostariophysi and Euteleosteomorpha), while Anguilliformes (Elopomorpha) appears as the earliest diverging branching lineage among teleosts. Siluriformes and Cypriniformes formed a monophyletic group (Ostariophysi), the sister group of Euteleosteomorpha (Acanthomorpha and Salmoniformes). A preliminary synthesis of ag-FSH was carried out in a specific HEK293F^TM^ cell line suspension culture, in order to ascertain that the cloned transcripts could express the heterodimeric hormone. Identity tests were also performed, leaving no doubt that we were in the presence of the heterodimeric ag-FSH. We must emphasize that no specific immunoassay, antibody or reference standard were available, which made our task quite difficult. Thanks to these results it will now be much easier to synthesize and identify ag-LH and possibly other hormones of this fish species.

In conclusion, this study characterizes the sequences of ag-FSHβ and ag-LHβ peptides, comparing them to those of other teleosts. The 3D models of ag-FSH and ag-LH provided interesting structural comparisons with those reported for other fish GTHs. Additional data for the phylogenetic relationship among Teleostei are provided but they need further confirmation by possibly adding also the ag-TSHβ sequence and using at least three different reconstruction methods (NJ, ML, MP or Bayesian) in order to determine the most reliable tree typology for each β-subunit. The GTHα of *A*. *gigas* characterized in a previous work and the sequences found here will be used for the biotechnological synthesis of *A*. *gigas* gonadotrophic hormones. This will be useful for checking the quaternary structures of ag-FSH and ag-LH and their binding to receptors, as well as for physiological and fertility studies and related applications, which are essential for the preservation of this important fish species.

## Supporting information

S1 Fig **Root mean squared deviation (RMSD) values of GTHα, FSHβ and LHβ subunits in the respective heterodimers (A); radius of gyration (Rg) of all three subunits in the complexes (B); hydrophobic and hydrophilic surface of two simulated hormones (C) and total number of intra and intermolecular hydrogen bonds of both simulated hormones along the MD simulation (D)**.
(PDF)

S2 Fig **Root mean squared fluctuation (RMSF) of the α-carbon of the α-subunits (A) and β-subunits (B) of both studied hormones**.
(PDF)

S3 Fig Comparison between the amino acid composition of seat-belts from ag-LH and ag-FSH (present work) and the gonadotropins studied by Aizen et al. [66]. Amino acids were classified as cysteines (pink), negatively charged (red), positively charged (blue), polar (cyan) and hydrophobic (orange)
(PDF)

S4 Fig Phylogenetic tree of concatenated FSHβ and LHβ DNA sequences obtained by the methods of maximum parsimony (bootstrap values below the branches) and maximum likelihood (bootstrap values above the branches). (PDF)

S1 Table Results of primary alignment obtained from HHPRED server. (PDF)

S2 Table Average physicochemical parameters obtained from 20ns of molecular dynamics simulation. (PDF)

S3 Table Results obtained from Prosa-web, Ramachandran plot and Verify 3D validations for both hormones. (PDF)
