# Supplementary material for: Molecular cloning and characterization of pirarucu (Arapaima gigas) follicle-stimulating hormone and luteinizing hormone β-subunit cDNAs
Source: PLoS One. 2017 Aug 28;12(8):e0183545. doi: 10.1371/journal.pone.0183545 (PMC5573580; doi:10.1371/journal.pone.0183545)

S1 Fig. Root mean squared deviation (RMSD) values of GTH α, FSH β e LH β subunities in its respective heterodimers (A); radius of gyration (Rg) of all three subunities in its complexes (B); hydrophobic and hydrophilic surface of two simulated hormones (C) and total number of intra and intermolecular hydrogen bonds of both simulated hormones along MD simulation (D).

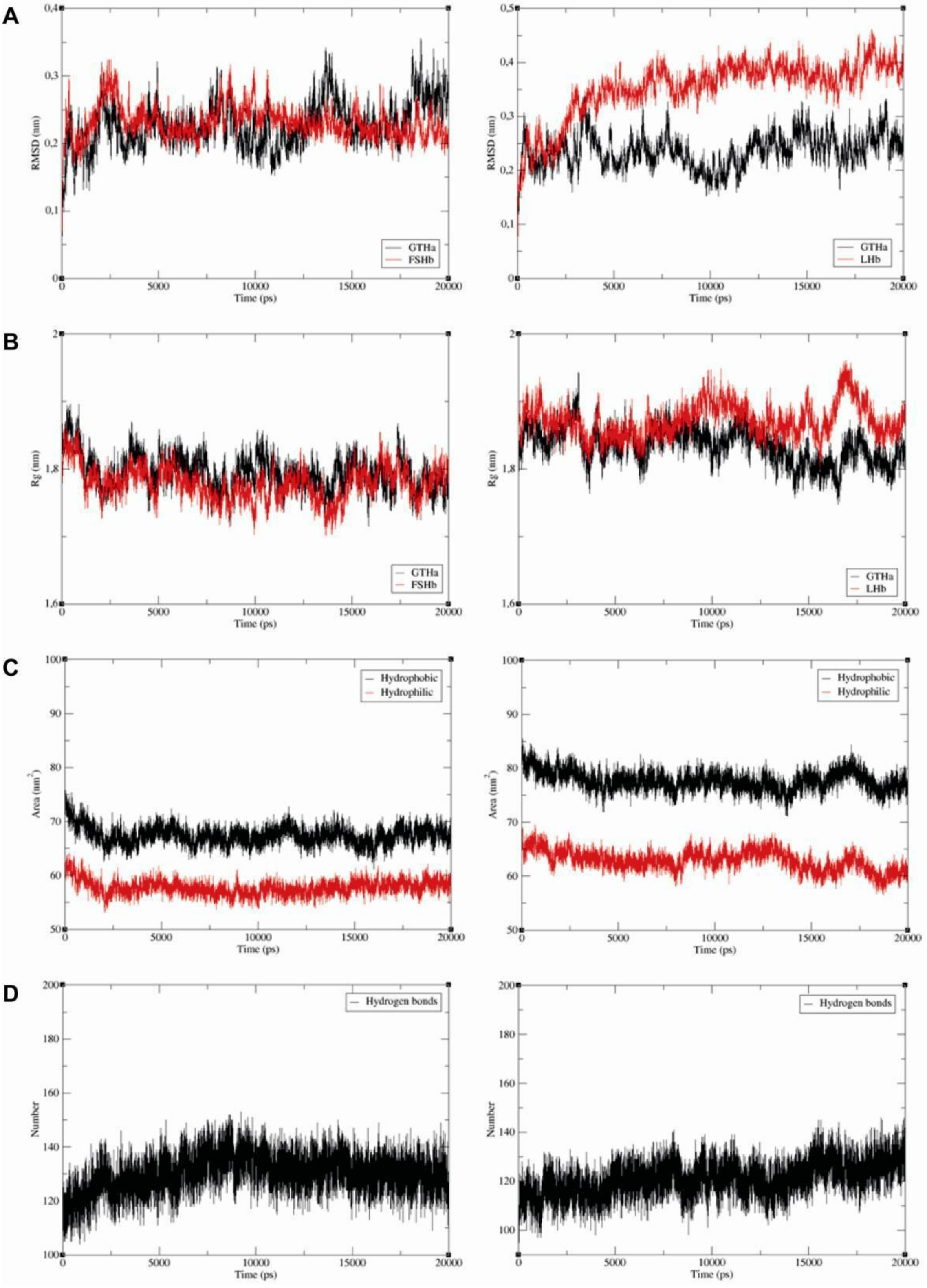

Supplement: S1 Fig — Root mean squared deviation (RMSD) values of GTHα, FSHβ and LHβ subunits in the respective heterodimers (A); radius of gyration (Rg) of all three subunits in the complexes (B); hydrophobic and hydrophilic surface of two simulated hormones (C) and total number of intra and intermolecular hydrogen bonds of both simulated hormones along the MD simulation (D). (PDF) [file pone.0183545.s001.pdf]
