# Supplementary material for: Molecular cloning and characterization of pirarucu (Arapaima gigas) follicle-stimulating hormone and luteinizing hormone β-subunit cDNAs
Source: PLoS One. 2017 Aug 28;12(8):e0183545. doi: 10.1371/journal.pone.0183545 (PMC5573580; doi:10.1371/journal.pone.0183545)

S2 Fig. Root mean squared fluctuation (RMSF) of α-carbon of α-subunits (A) and β-subunits (B) of both studied hormones

A

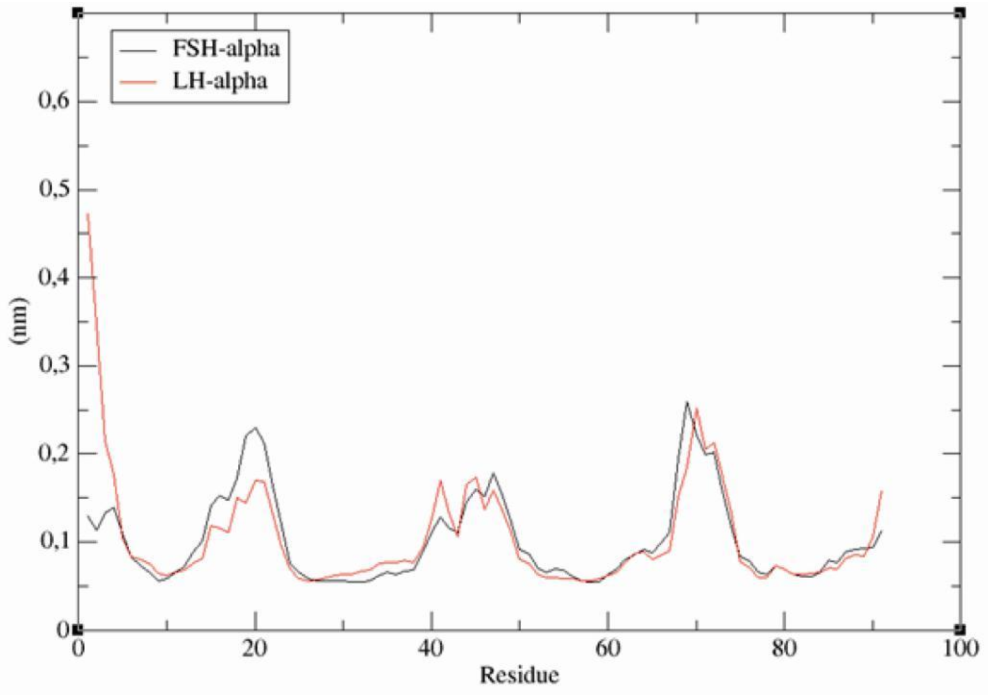

B

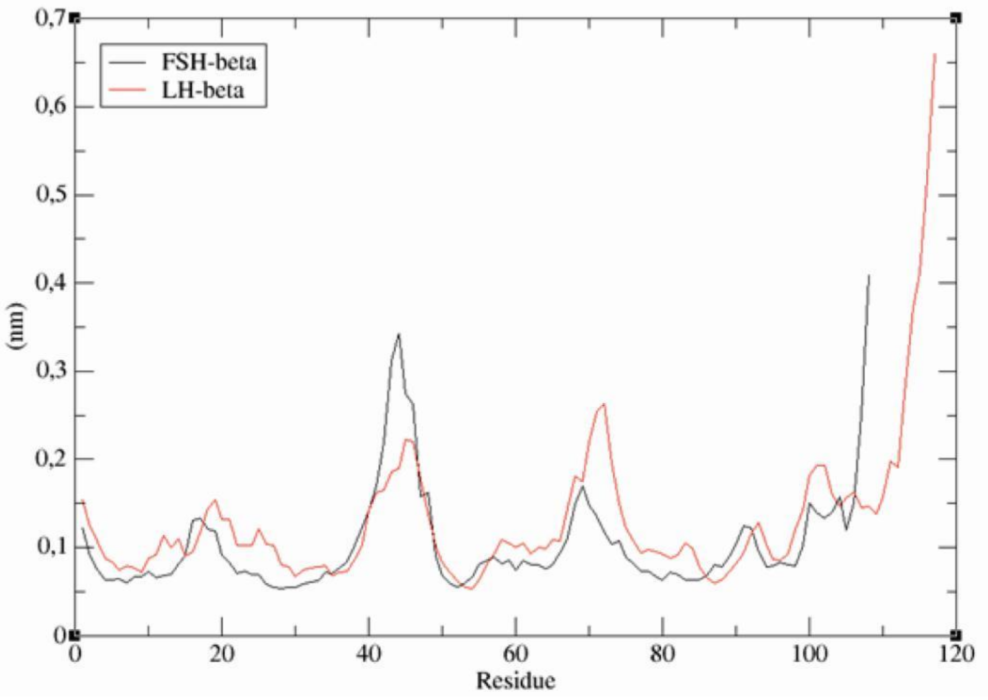

Supplement: S2 Fig — Root mean squared fluctuation (RMSF) of the α-carbon of the α-subunits (A) and β-subunits (B) of both studied hormones. (PDF) [file pone.0183545.s002.pdf]
