# Supplementary material for: Molecular cloning and characterization of pirarucu (Arapaima gigas) follicle-stimulating hormone and luteinizing hormone β-subunit cDNAs
Source: PLoS One. 2017 Aug 28;12(8):e0183545. doi: 10.1371/journal.pone.0183545 (PMC5573580; doi:10.1371/journal.pone.0183545)

S3 Fig. Comparison of seat-belt aminoacid composition between agLH and agFSH and gonadotropins used in the work of Aizen et al. 2012. Aminoacids were classified as cysteines (pink), negativelly charged (red), positivelly charged (blue), polar (cyan) and hydrophobic (orange).

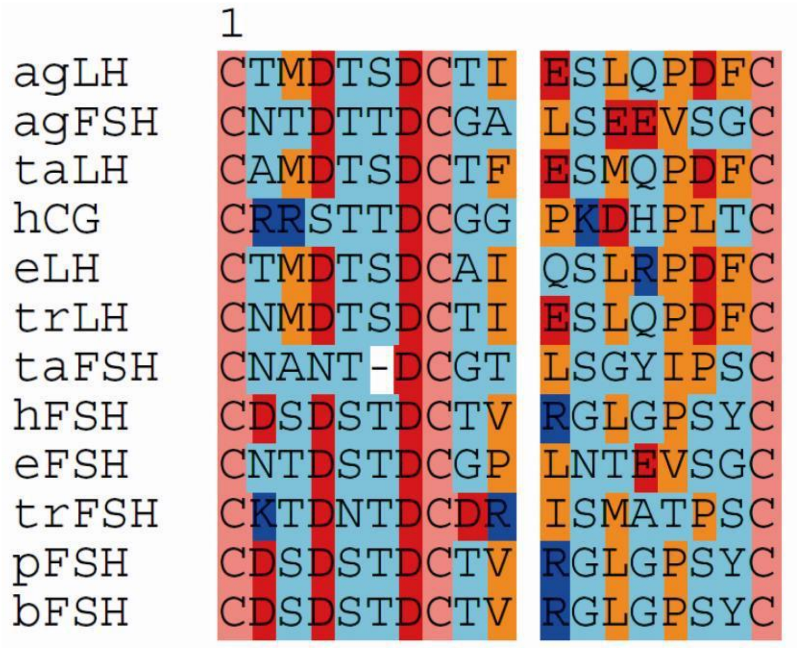

Supplement: S3 Fig — Amino acids were classified as cysteines (pink), negatively charged (red), positively charged (blue), polar (cyan) and hydrophobic (orange) (PDF) [file pone.0183545.s003.pdf]
