# Supplementary material for: Molecular cloning and characterization of pirarucu (Arapaima gigas) follicle-stimulating hormone and luteinizing hormone β-subunit cDNAs
Source: PLoS One. 2017 Aug 28;12(8):e0183545. doi: 10.1371/journal.pone.0183545 (PMC5573580; doi:10.1371/journal.pone.0183545)

S4 Fig. Phylogenetic tree of concatenated *fshb* and *lhb* DNA sequences obtained by the methods of Maximum Parsimony (bootstrap values below the branches) and Maximum Likelihood (bootstrap values above the branches)

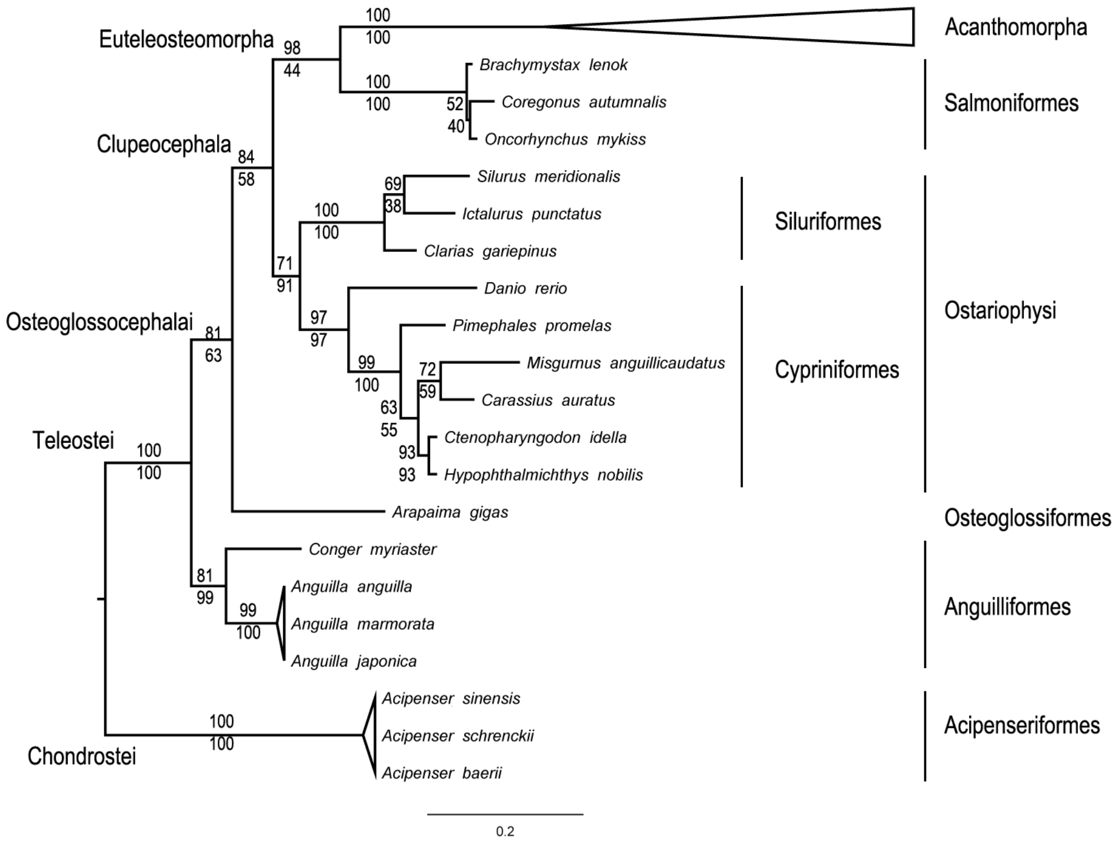

Supplement: S4 Fig — (PDF) [file pone.0183545.s004.pdf]
