# Supplementary material for: Molecular cloning and characterization of pirarucu (Arapaima gigas) follicle-stimulating hormone and luteinizing hormone β-subunit cDNAs
Source: PLoS One. 2017 Aug 28;12(8):e0183545. doi: 10.1371/journal.pone.0183545 (PMC5573580; doi:10.1371/journal.pone.0183545)
Supplement: S1 Table — (PDF) [file pone.0183545.s005.pdf]

S1 Table. Results of primary alignment obtained from HHPRED server.

Model	Template	Identity	Probability	E value	Score
**GTHα**	4ay9_a	68%	100%	$2.6x10^{-67}$	392.95
**FSHβ**	4ay9_b	47%	100%	$7.3x10^{-51}$	302.77
**LHβ**	1hcn_b	48%	100%	$5.3x10^{-50}$	298.06
