# Supplementary material for: Molecular cloning and characterization of pirarucu (Arapaima gigas) follicle-stimulating hormone and luteinizing hormone β-subunit cDNAs
Source: PLoS One. 2017 Aug 28;12(8):e0183545. doi: 10.1371/journal.pone.0183545 (PMC5573580; doi:10.1371/journal.pone.0183545)
Supplement: S2 Table — (PDF) [file pone.0183545.s006.pdf]

S2 Table. Average physicochemical parameters obtained from 20ns of molecular dynamics simulation

	**FSH**	**LH**
**Total Energy**	-876859 ± 31 kcal/mol	-880759 ± 10 kcal/mol
**Temperature**	309.996 ± 0.002 K	309.993 ± 0.003 K
**Pressure**	1.006 ± 0.150 bar	1.208 ± 0.200 bar
**Density**	979.808 ± 0.022 kg/m^3	979.993 ± 0.034 kg/m^3
**Volume**	783.487 ± 0.017 nm^3	789.462 ± 0.027 nm^3
