# Supplementary material for: Molecular cloning and characterization of pirarucu (Arapaima gigas) follicle-stimulating hormone and luteinizing hormone β-subunit cDNAs
Source: PLoS One. 2017 Aug 28;12(8):e0183545. doi: 10.1371/journal.pone.0183545 (PMC5573580; doi:10.1371/journal.pone.0183545)
Supplement: S3 Table — (PDF) [file pone.0183545.s007.pdf]

S3 Table. Results obtained from Prosa-web, Ramachandran plot and Verify 3D validations for both hormones.

	FSH	
	**α subunit**	**β subunit**
Prosa	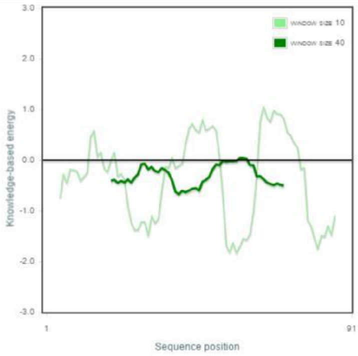	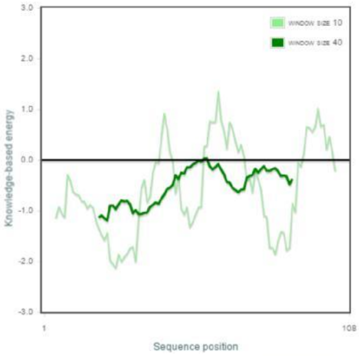
Ramachandran plot	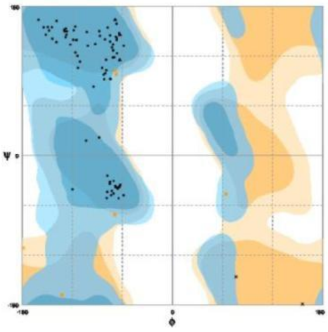	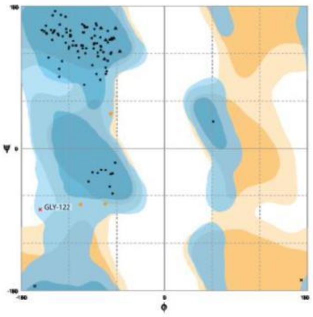
Ramachandran outliers*	0%	0%
Verify 3D (score < 0)	0 residues	0 residues
	**LH**	
	**α subunit**	**β subunit**
Prosa	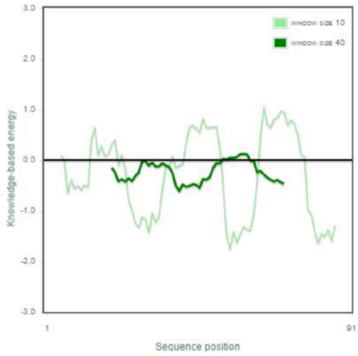	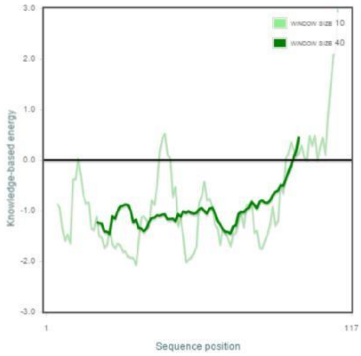
Ramachandran plot	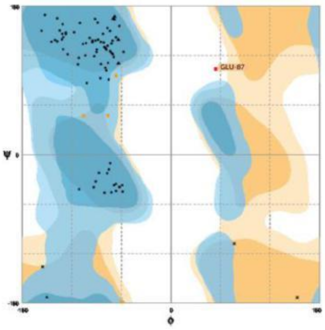	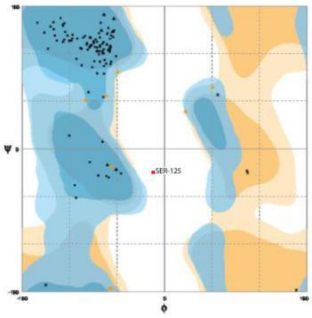
Ramachandran outliers*	1.1%	0.9%
Verify 3D (score < 0)	0 residues	4 residues + C terminal

*GLY residues were not considered outliers
